# Supplementary material for: Superconducting spintronic tunnel diode
Source: Nat Commun. 2022 May 4;13:2431. doi: 10.1038/s41467-022-29990-2 (PMC9068691; doi:10.1038/s41467-022-29990-2)
Supplement: Supplementary file 1 — Supplementary Information [file 41467_2022_29990_MOESM1_ESM.pdf]

Supplementary Information: Superconducting spintronic tunnel diode

E. Strambini,[1,*] M. Spies,[1,†] N. Ligato,[1] S. Ilic,[2] M. Rouco,[2] Carmen González Orellana,[2] Maxim Ilyn,[2] Celia Rogero,[2,3] F.S. Bergeret,[2,3] J. S. Moodera,[4] P. Virtanen,[5] T. T. Heikkilä,[5] and F. Giazotto[1,‡]

[1]*NEST, Istituto Nanoscienze-CNR and Scuola Normale Superiore, I-56127 Pisa, Italy*

[2]*Centro de Física de Materiales (CFM-MPC) Centro Mixto CSIC-UPV/EHU, E-20018 Donostia-San Sebastián, Spain*

[3]*Donostia International Physics Center (DIPC), 20018 Donostia–San Sebastián, Spain*

[4]*Physics Department and Plasma Science and Fusion Center, Massachusetts Institute of Technology, Cambridge, Massachusetts 02139, USA*

[5]*Department of Physics and Nanoscience Center, University of Jyväskylä, P.O. Box 35 (YFL), FI-40014 University of Jyväskylä, Finland*

[*] elia.strambini@sns.it

[†] maria.spies@nano.cnr.it

[‡] francesco.giazotto@sns.it

I. ANALYSIS OF THE DIODE IN CURRENT BIAS.

Starting from the $I(V)$ characteristics shown in Fig. 2b of the main text, it is possible to quantify the rectification efficiency when operating the diode in current bias. Notably, due to the anti-symmetric, non-linear response of the diode, this is not simply equivalent to the voltage bias analysis shown in the main text as one can see comparing it with Fig. S1. In fact, the high resistance of the tunnel junction at low voltages promotes a sudden response of the anti-symmetric voltage (red curve in Fig. S1a) while the symmetric component grows at larger current bias (black curve in Fig. S1a). As a result, the rectification in current bias is smaller, with a maximum rectification of $\sim 20\%$ as shown in Fig. S1b.

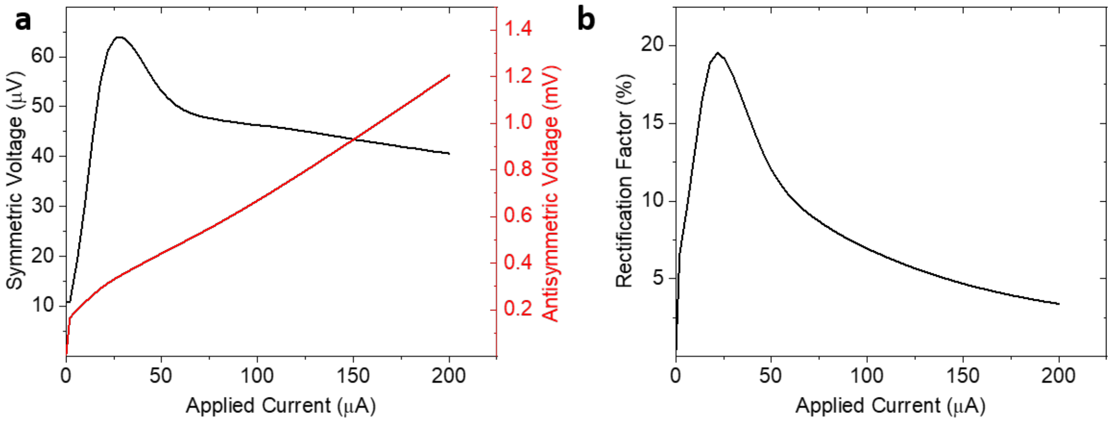

FIG. S1. **a**, Symmetric and anti-symmetric parts ($V_{Sym} = (V(I) + V(-I))/2$, $V_{Antisym} = (V(I) - V(-I))/2$) of the $V(I)$ characteristic equivalent to the one shown in Fig. 2b of the main text. **b**, Rectification factor ($R = V_{Sym}/V_{Antisym}$) evaluated for this configuration. The maximum rectification occurs at low currents and reaches around $\sim 20\%$ there.

II. ADDITIONAL SAMPLE STRUCTURES.

Besides the superconductor/ferromagnetic insulator/normal metal (S/FI/N) structure shown in the main text, different material combinations with equivalent spin-filtering and spin-splitting have been investigated. Most notably, a FI/S/I/F structure (where I is an insulator and F is a metallic ferromagnet) have been investigated. Differing from S/FI/N junctions, here, the spin-filtering and spin-splitting are decoupled. The former is still provided by the FI/S interface, while the latter is due to the I/F tunnel barrier.

Samples are cross-bars made by electron-beam evaporation employing an in-situ shadow mask on a substrate of fused silica and consist of layers of EuS(14 nm)/ Al(9 nm)/ AlO_x(4-5 nm)/ Co(10 nm)/ CaF(7 nm). The overlap between the Al and the Co strip has an area of 300×300 μm^2. The tunneling spectroscopy is carried out at cryogenic temperatures down to 50 mK in a filtered cryogen-free dilution refrigerator. The $I(V)$ characteristics are obtained from DC four-wire measurements as described in the main text.

The data analysis on the $I(V)$ characteristic at $B = 0$ is shown in Fig. S2. Notably, as shown in Fig. S2a and b, in this device the zero-bias conductance is more pronounced with respect to the the S/FI/N sample shown in the main text. On the other hand, large spin-splitting and spin-filtering are visible even at zero magnetic field thanks to the stronger ferromagnetism of the EuS layer. Therefore, even if the rectification is smaller with respect to the S/FI/N devices (here the maximum rectification is $\sim 18\%$ as estimated in Figs. S2d and f) the presence of a sizable rectification, even in the absence of an external magnetic field, makes it appealing for applications. Moreover, differing from S/FI/N junctions where the direction of the diode is fixed by the sign of the exchange interactions at the EuS/Al interface, in this typology of device the direction of the diode can be inverted by changing the relative magnetization of the FI and F layers (parallel or anti-parallel), introducing additional functionalities.

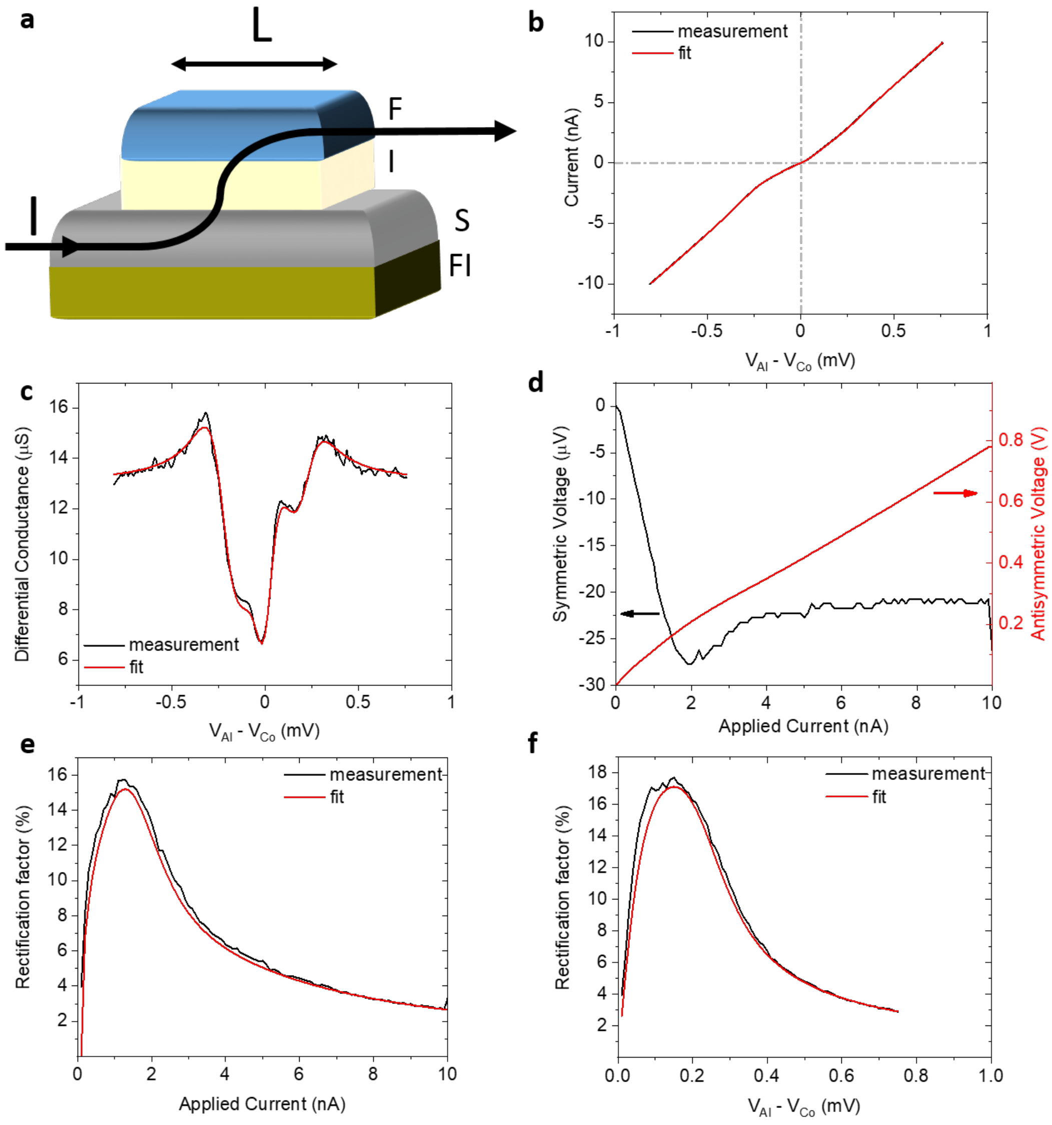

FIG. S2. **Rectification of a superconducting diode made with alternative materials: a FI/S/I/F junction.** **a**, Schematic of the tunnel junction. The path of the tunneling current is indicated by the black line and its arrows. **b**, Current-to-Voltage ($I(V)$) characteristic of the junction measured at $T \simeq 50\,\text{mK}$, $B = 0\,\text{T}$. **c**, Differential conductance obtained from the numerical derivative of **b**. The fits in **b** and **c** have been obtained from Eq. (3) and (14) of the main text with the following parameters: $\Delta_0 = 0.228$ meV, $h = 0.097$ meV, $P = 0.3$, $\Gamma = 0.01$ meV, $T = 250$ mK, $\tau_{sf} = 15$ meV^{-1}, $\tau_{so} = 600$ meV^{-1}. **d**, Symmetric and anti-symmetric parts of the $I(V)$ characteristic in **b** showing a sizable symmetric component of the voltage. **e** and **f**, Rectification coefficients evaluated from the $I(V)$ characteristics in current and voltage bias, respectively (black line). The comparison of the rectification extracted from the full numerical model is also shown (red line).

III. APPLICATIONS FOR DETECTION.

A. Resolution and Noise equivalent Power

The sizable rectification of the superconducting tunnel diode observed both in the direct (i) and in the transverse (ii) configuration can find an immediate application in the detection of electromagnetic radiations. Starting from the characterizations presented in the main text, it is possible to estimate the maximum resolution and the noise equivalent power (NEP) of a detector based on this technology. For configuration (i) the DC response to a sinusoidal low-frequency ($\hbar\omega \ll \Delta$) AC signal ($V_{AC} = V_0 \sin(\omega t)$) can be estimated by averaging the current response over the signal time period T:

$$I_{DC} = \int_{-T/2}^{T/2} I(V_{AC}) \frac{dt}{T} = \int_{-T/2}^{T/2} I_{Sym}(V_{AC}) \frac{dt}{T}, \tag{S1}$$

and the resulting power dissipated by the signal reads:

$$P = \int_{-T/2}^{T/2} V_{AC}\, I(V_{AC}) \frac{dt}{T} = \int_{-T/2}^{T/2} V_{AC}\, I_{Antisym}(V_{AC}) \frac{dt}{T}. \tag{S2}$$

In Fig. S3a we show the $I_{DC}(P)$ estimated from the latter equations applied to the $I(V)$ characteristic of the superconducting diode in the direct configuration presented in Fig. 2b of the main text. The resulting transfer function (dI_{DC}/dP) is shown in Fig. S3b and determines the resolution of the detector. If terminated with a low-noise current amplifier with an input noise of $\sim 0.2\,\mathrm{fA}/\sqrt{\mathrm{Hz}}$ (e.g. FEMTO LCA-2-10T as a conventional room-temperature amplifier) the detector can provide a very low NEP down to $\sim 1 \times 10^{-19}\,\mathrm{W}/\sqrt{\mathrm{Hz}}$ at low powers (see red solid line in Fig.S3c for the full power spectrum at 0.1 T and 0.02 K), already competing with state-of-the-art detectors. A high sensitivity is, however, preserved even at zero magnetic field, with a NEP of $\sim 1 \times 10^{-18}\,\mathrm{W}/\sqrt{\mathrm{Hz}}$ at low power (<pW) increasing up to $\sim 10^{-16}$ at few nW (see red dashed line in Fig.S3c). A similar monotonic degradation applies to higher temperatures (up to 1.9 K) at 0.1 T with a NEP of $10^{-17} \sim 1\,\mathrm{W}/\sqrt{\mathrm{Hz}}$ (see cyan and magenta lines in Fig.S3c). As discussed below, these estimates show how it is possible to reach a regime where amplifier noise does not determine the device sensitivity.

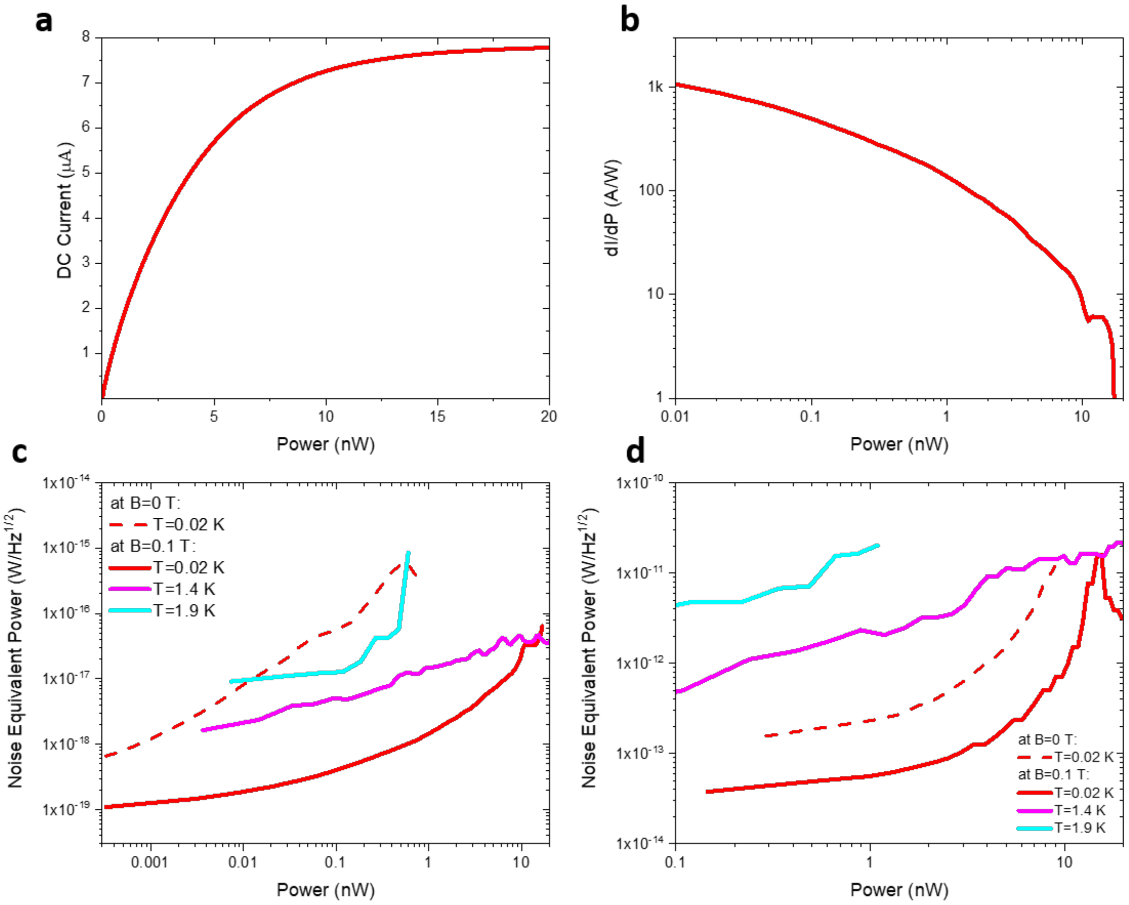

FIG. S3. **Resolution and NEP of the N/FI/S junction in the direct configuration.** **a**, DC rectified current (I_{DC}) vs. the power of the input AC signal evaluated from the $I(V)$ characteristic in Fig. 2b and Eq. S1, S2 in the closed circuit configuration at 20 mK with an external magnetic field of 0.1 T. **b**, Power spectrum of the transfer function (dI/dP) resulting from **a**. **c**, Power spectrum of the NEP evaluated by the ratio between the input noise of a room temperature current amplifier (FEMTO LCA-2-10T) $\sim 0.2\,\mathrm{fA}/\sqrt{\mathrm{Hz}}$ and the transfer function in **b**. The estimations have been done for different magnetic fields and temperatures as indicated in the legend. **d**, NEP evaluated for the open circuit configuration, starting from Eq. S3 and S4, with an input noise voltage of $\sim 0.4\,\mathrm{nV}/\sqrt{\mathrm{Hz}}$ (amplifier: DLPVA-100-BUN-S). The estimations have been done for different magnetic fields and temperatures as indicated in the legend.

Fig.S3d shows the same evaluations in an open circuit configuration where the rectified DC voltage and power are quantified in a similar mode:

$$V_{DC} = \int_{-T/2}^{T/2} V(I_{AC}) \frac{dt}{T} = \int_{-T/2}^{T/2} V_{Sym}(I_{AC}) \frac{dt}{T}, \tag{S3}$$

$$P = \int_{-T/2}^{T/2} I_{AC} \, V(I_{AC}) \frac{dt}{T} = \int_{-T/2}^{T/2} I_{AC} \, V_{Antisym}(I_{AC}) \frac{dt}{T}. \tag{S4}$$

Here $I_{AC} = I_0 \sin(\omega t)$ is the AC signal to probe. Here, the resulting NEP evaluated with an input noise voltage of $\sim 0.4\,\mathrm{nV}/\sqrt{\mathrm{Hz}}$ is significantly smaller ($\sim 1 \times 10^{-14}\,\mathrm{W}/\sqrt{\mathrm{Hz}}$), probably due to the small impedance of the tunnel junction favoring the detection in the closed circuit configuration.

In the transverse configuration (ii), the rectification response can be estimated from Eq. S3 in a similar way, starting from the $V_{sym}(I_{Cu})$ characteristics shown in Fig. 3 and Fig. 4 of the main text. Differing from configuration (i), the power will be mainly dissipated in the Cu strip and can be estimated from its simple Ohmic response:

$$P = \int_{-T/2}^{T/2} I_{AC}^2 \, R \frac{dt}{T} = \frac{I_0^2 \, R}{2}, \tag{S5}$$

where $R \simeq 2\Omega$ is the lateral resistance of the Cu lead at the interface with the EuS.

In Fig. S4a and d, the $V_{DC}(P)$ is shown for different temperatures and magnetic fields, respectively. The resulting resolutions dV_{DC}/dP are shown in Fig. S4b and e. The NEP estimated with an input noise of $\sim$ $0.4\,\mathrm{nV}/\sqrt{\mathrm{Hz}}$ (e.g. DLPVA-100-BUN-S as a room-temperature voltage amplifier) is shown in Fig. S4c and f. Notably, in this configuration the NEP of $\sim$ 10^{-11} - 10^{-12} $\mathrm{W}/\sqrt{\mathrm{Hz}}$ is one order of magnitude larger with respect to the NEP evaluated in configuration (i) for the same open circuit configuration (Fig. S3d). On the other hand, in this configuration the detector is sensitive to a larger range of powers (up to 120 nW with no sign of saturation) and the DC signal originated across the junction is already decoupled from the AC component flowing in the Cu strip. Note that the high NEP here partially results from the fact that a large part of the power is "wasted" in heating Cu that has a strong electron-phonon coupling compared to the superconducting Al. A better alternative would be to heat Al, but that would require frequencies larger than the gap ($\hbar\omega > 2\Delta$). Such frequencies are not directly accessible in our setup.

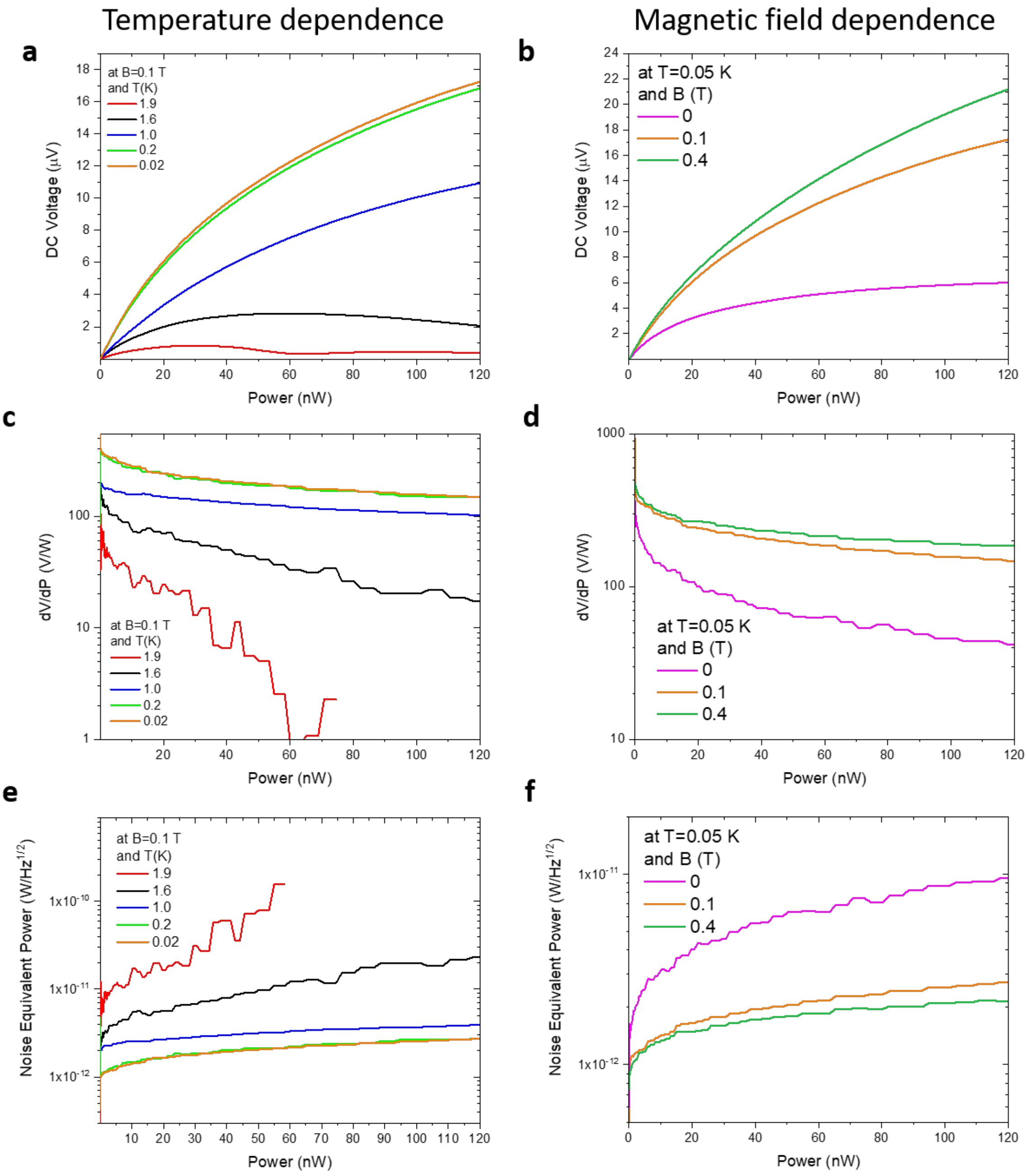

FIG. S4. **Resolution and NEP of the N/FI/S junction in the transverse configuration.** **a**, DC rectified voltage vs. the power of the input AC signal estimated in the transverse open-circuit configuration at different temperatures and **b**, magnetic fields. **c**, Power spectrum of the transfer function (dV/dP) resulting from **a**. The equivalent power spectrum resulting from **b** is shown in **d**. In panel **e**, the power spectrum of the NEP evaluated from **c** is shown for an input noise voltage of $\sim 0.4\,\mathrm{nV}/\sqrt{\mathrm{Hz}}$ (amplifier: DLPVA-100-BUN-S). The equivalent power spectrum resulting from **d** is shown in **f**.

In Fig. S5 we estimate the resolution and NEP in direct configuration (i) for the additional sample structure FI/S/I/F introduced earlier ($I(V)$ shown in Fig. S2). Thanks to the strong ferromagnetism of this device even at no applied external magnetic field the NEP reaches an impressive $\sim 10^{-18}$ - 10^{-19} W/$\sqrt{\text{Hz}}$, but only for low powers due to the higher impedance (four orders of magnitude) of the tunnel junction. Such a high impedance improves the NEP in the closed circuit configuration reaching values of $\sim 10^{-17}$ - 10^{-18} W/$\sqrt{\text{Hz}}$, which is much smaller then the N/FI/S counterpart.

B. Intrinsic noise for a rectifier detector

The above values for the Noise Equivalent Power are the amplifier contributions to the total NEP. In good detectors they should be below the intrinsic noise values. Let us consider the case where configuration (i) is used as a direct detector, where the incoming radiation power with frequency $\omega \gg \Delta$ is rectified to give the dc current that provides the detector response. Following [1], the dc current in this regime is given by

$$I_{\text{dc}} = \frac{Peh}{\omega^2} P_{\text{ac}} \equiv \lambda_I P_{\text{ac}}. \tag{S6}$$

The intrinsic noise is due to the thermal Johnson-Nyquist noise across the junction, with zero-frequency spectral density

$$S_I = 4k_B TG \tag{S7}$$

and using the linear junction conductance G. The intrinsic NEP is obtained from

$$NEP = \frac{\sqrt{S_I}}{\lambda_I} = \frac{2\omega^2\sqrt{k_B TG}}{Peh} \tag{S8}$$

$$= \frac{2\tilde{\omega}^2}{P\tilde{h}}\sqrt{tg}\frac{\Delta^{3/2}}{(0.2\text{meV})^{3/2}} \cdot 5 \cdot 10^{-15}\ \frac{\text{W}}{\sqrt{\text{Hz}}}. \tag{S9}$$

Here $\tilde{\omega} = \omega/\Delta$, $\tilde{h} = h/\Delta$, $t = k_B T/\Delta$ and $g = G{\cdot}50\ \Omega$. Note that this does not include the contribution from non-ideal quantum efficiency.

For example, with $\omega = 3\Delta$, $k_B T = 0.1\Delta$, $h = 0.2\Delta$, $P = 0.5$, $G = 2$ mS and $\Delta = 0.2$ meV, the intrinsic NEP would be about 5×10^{-16} W/$\sqrt{\text{Hz}}$.

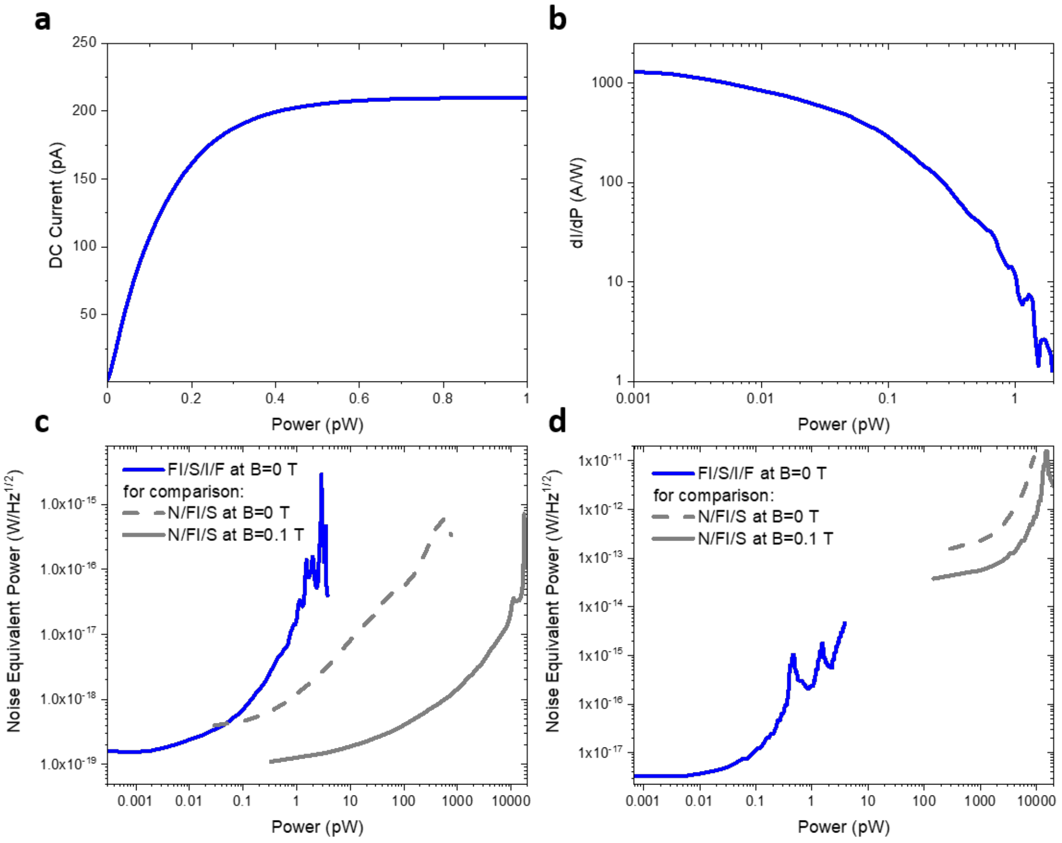

FIG. S5. **Resolution and NEP of the FI/S/I/F junction in the direct configuration.** **a**, DC rectified current (I_{DC}) vs. the power of the input AC signal evaluated from the $I(V)$ characteristic in Fig. S2b and Eq. S1, S2 in the closed circuit configuration at 50 mK and zero magnetic field. **b**, Power spectrum of the transfer function (dI/dP) resulting form **a**. **c**, Power spectrum of the NEP evaluated by the ratio between the input noise of a room temperature current amplifier (FEMTO LCA-2-10T) $\sim 0.2\,\mathrm{fA}/\sqrt{\mathrm{Hz}}$ and the transfer function in **b**. A comparison with the NEP evaluated for the N/FI/S junction is also shown (gray lines) **d**, NEP evaluated for the open circuit configuration, starting from Eq. S3 and S4, with an input noise voltage of $\sim 0.4\,\mathrm{nV}/\sqrt{\mathrm{Hz}}$ (amplifier: DLPVA-100-BUN-S). A comparison with the NEP evaluated for the N/FI/S junction is also shown (gray lines).

IV. MODELING CONTRIBUTIONS OF RECTIFICATION AND THERMOVOLTAGE.

A. "Direct" configuration (i).

To model heating effects in the "direct" configuration (i), we apply the thermal model

$$\dot{Q}_{S,\mathrm{eph}}(T_S, T_{ph}) = \dot{Q}_{S,\mathrm{tun}}(T_S, T_N, V)\,, \tag{S10a}$$

$$\dot{Q}_{N,\mathrm{eph}}(T_S, T_{ph}) = \dot{Q}_{N,\mathrm{tun}}(T_S, T_N, V) + P_{N,\mathrm{Joule}}\,, \tag{S10b}$$

where we include electron-phonon thermal relaxation, the heat flow and Joule heat associated with tunneling, and Joule heat dissipated on the normal side. In the parameter range considered, heat diffusion out of the large-area junction is negligible.

Expressions for $\dot{Q}_{\mathrm{eph}}$ and $\dot{Q}_{\mathrm{tun}}$ can be found in Ref. [2]. The Joule power dissipated on the N-side we approximate with $P_{N,\mathrm{Joule}} \approx R_{\mathrm{Cu}} I^2$ where $R_{\mathrm{Cu}} \approx \rho_{\mathrm{Cu}} L_x/(W t_{\mathrm{Cu}})$ is the lateral resistance of the copper under junction of size $L_x \times W$ with layer thickness t_{Cu}.

We for simplicity neglect here non-uniformity in the current distribution and temperature in the junction, which is accurate for $R_T \gg R_{\mathrm{Cu}}$. Here $R_T \sim 2R_{\mathrm{Cu}}$, which is sufficient for estimating the relative order of magnitude of the effects. The non-uniformity is important for configuration (ii), which we discuss separately below.

The resulting temperatures T_N and T_S are shown in Fig. S6. There is generally substantial heating on the S side, because most of the power is dissipated as Joule heat in the junction. Large part of this enters the S side, which has both smaller volume and smaller e-ph coupling than the N side.

The resulting symmetric and antisymmetric components of the IV curve are shown in Fig. S7. The symmetric component evaluated at $V = 0$ but with $T_S \neq T_N$ comes solely from the thermoelectric effect, and gives an estimate for its magnitude. Generally, we can observe that in the regime where the total effect is largest, rectification dominates, with thermoelectric current being $\sim$ 20%–50% of it and having an opposite sign.

The precise results however depend somewhat on the thermal model, and the model here is likely not fully accurate. In the main text, we do not include heating and rather concentrate on the dominating rectification effect.

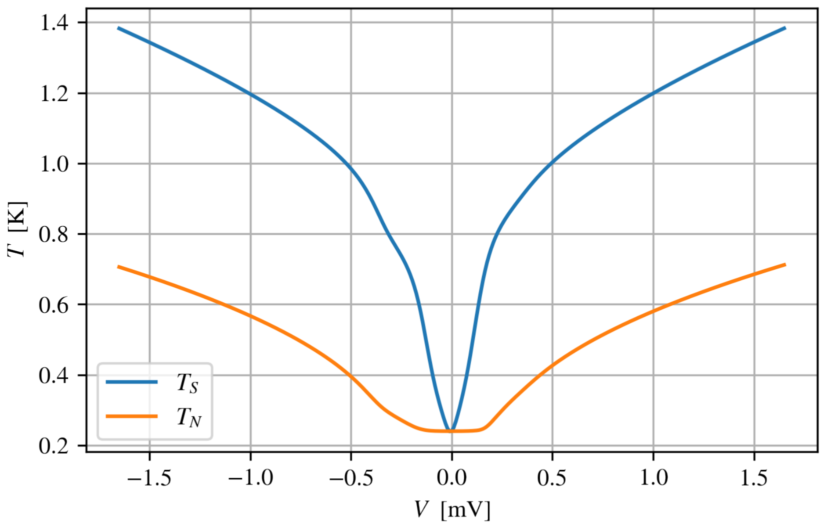

FIG. S6. Temperatures T_N, T_S obtained from thermal model (S10), for the "direct" configuration (i).

B. Lateral configuration (ii).

To obtain input parameters for the modeling including the thermoelectric effects, we have fitted the IV data sets in the lateral configuration (ii) with the following model:

$$\frac{dI_{\rm expt}}{dV}(V_i, V_{H,j}) \sim G_T \frac{d\tilde{I}_{\rm model}}{dV}(aV_{H,j}, V_i + V_{{\rm off},j}, T_{N,j}) \,, \tag{S11}$$

where G_T, a, $V_{{\rm off},j}$ and $T_{N,j}$ are the fit parameters, corresponding to a set of values V_i and $V_{H,j}$ for the bias and heating voltages, and $dI_{\rm expt}/dV$ the observed differential conductance. The lateral resistance is $R_x = aR_H$, where $R_H \approx 4.2\,{\rm k\Omega}$ is the resistance relating the heating voltage to the heating current, $I_H = V_H/R_H$. The theoretical current model is

$$\frac{d\tilde{I}_{\rm model}}{dV}(V', V, T_N) = \int_{-1/2}^{1/2} ds\, G_{T,0}^{-1} \frac{dI_{NFIS}}{dV}(V + sV', T_N, T_S) \,, \tag{S12}$$

where $I_{NFIS}(V, T_N, T_S)$ is the current-voltage relation discussed in Ref. [3]. We include the effects of Γ and other parameters affecting the density of states of the superconductor as in the main text, determined by separate fits done for $V_H = 0$. We assume the order parameter Δ remains roughly constant in the parameter range considered, in which case the differential conductance is independent of the superconductor temperature T_S.

After obtaining the above parameters, we find the temperature T_S of the superconducting

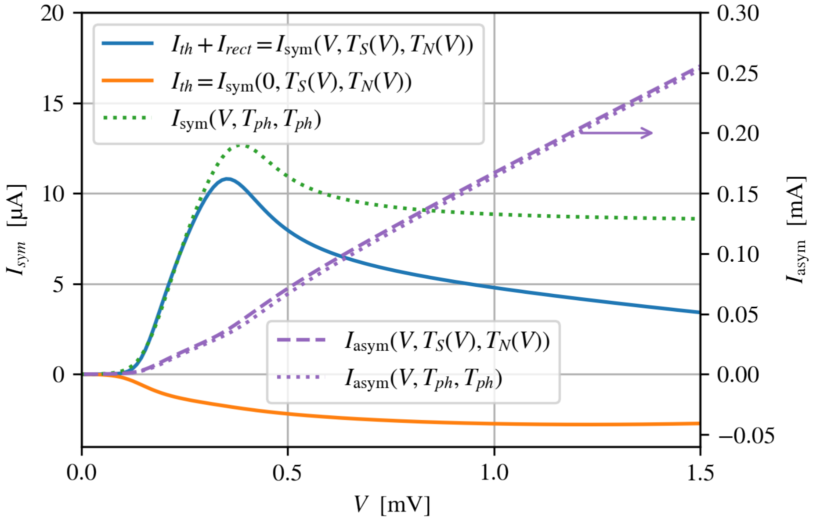

FIG. S7. Symmetric $I_{\rm sym}$ and antisymmetric $I_{\rm asym}$ components of the current, computed assuming the thermal model (S10). Estimate for the thermoelectric contribution is given by $I_{\rm th} \approx I_{\rm sym}(V = 0, T_S, T_N)$. Results at electronic bath temperature $T_{\rm ph} = 240$ mK without temperature differences are also shown for comparison.

side by solving the thermal balance model

$$\dot{Q}_{\rm tun}(V, T_N, T_S) = \dot{Q}_{\rm e-ph}(T_N, T_S)\,, \tag{S13}$$

where $\dot{Q}_{\rm tun}$ is the tunneling heat current to S obtained analogously as in Eq. (S12) (see Ref. [3]). It is balanced by electron-phonon relaxation, with heat current $\dot{Q}_{\rm e-ph}$ as described in Ref. [2], using literature parameters for Aluminum electron-phonon coupling [4], and including the effects from spin splitting, Γ and spin-flip scattering. The resulting T_S is shown in Fig. S8.

With the parameters so obtained, we find the open-circuit voltage $V = V_N - V_S$ in the superconductor by solving

$$0 = \int_{-1/2}^{1/2} ds\, I_{NFIS}(V + sV', T_N, T_S) \tag{S14}$$

and extract the part symmetric under inverting V_H. This can be calculated with and without thermoelectric effects, i.e., determining T_S from the heating model or setting $T_S = T_N$. Example of such calculations is shown in Fig. S9. We can observe that the magnitude of thermoelectricity predicted by this model is in the range of 25%–50%, supporting the result

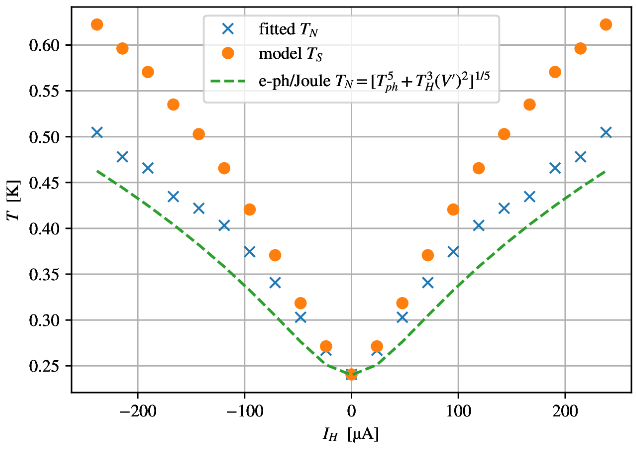

FIG. S8. Temperature of the normal side T_N obtained by fits to dI/dV measurements, and the temperature T_S obtained from Eq. (S13). Result from a local electron-phonon vs. Joule heating model for T_N, $\Sigma(T_N^5 - T_{\rm ph}^5) = \rho j_H^2$, is also shown for comparison.

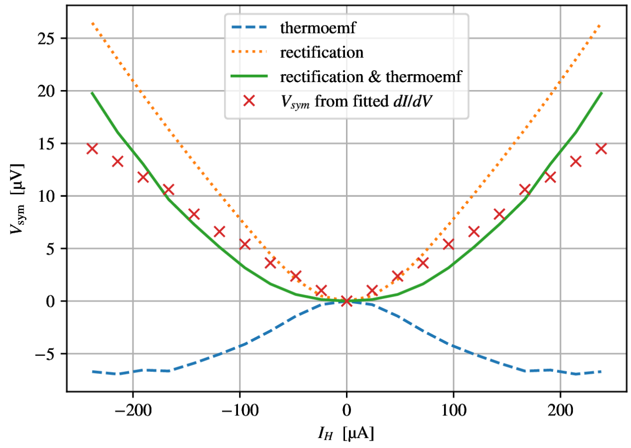

FIG. S9. Symmetric part of the open-circuit voltage $V = V_N - V_S$ in the superconductor, modeled based on the IV data at $B = 200\,\mathrm{mT}$.

obtained in an alternative way in the main text, even though the quantitative agreement is not fully complete. Moreover, we observe that the model predicts the two contributions to the symmetric voltage have opposite signs. This occurs because the heating model predicts $T_S > T_N$, since the electron-phonon coupling of Aluminum is strongly suppressed by superconductivity, and a part of the heating current tunnels in and out of the superconductor imparting direct Joule heating on it.

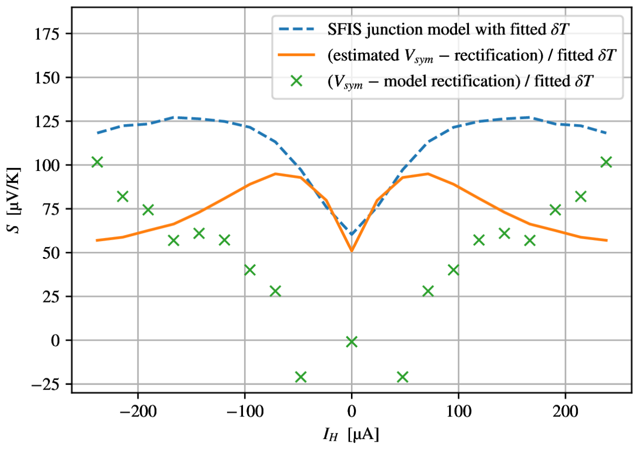

FIG. S10. Estimates for Seebeck coefficient. Obtained based on the temperatures in Fig. S8, and voltage from (i) computed from the junction model, (ii) dashed curve in Fig. S9, (iii) subtracting the rectification model from V_{sym} extracted from dI/dV measurements.

Based on the temperature difference obtained from the model, we show in Fig. S10 the Seebeck coefficient corresponding to the voltages in Fig. S9 and temperatures in Fig. S8. These results are all based on subtracting the counterfactual model result including only rectification, and hence the accuracy is limited to providing rough guidance of the likely order of magnitude.

Finally, we can note that the relative strength of the rectification and thermoelectricity varies depending on the junction length L_x. If the junction is very short, there is no transverse voltage drop or rectification, whereas if the junction is very long the rectification is large. We can estimate the length scale on which thermoelectricity starts to dominate as follows.

First, from characteristics of I_{NFIS} one can observe the rectification scales with the dimensionless parameter $\sim V'/\Delta$ describing the transverse voltage. On the other hand, thermoelectricity scales with $\sim \delta T/T$ where $\delta T = T_N - T_S$ is the temperature difference. For thermoelectricity to be large and dominating, we then want to simultaneously have $\delta T/T \sim 1$ and $eV' \ll \Delta$. In a rough estimate, under such conditions, the heat balance

equation (S13) can be approximated with

$$\frac{k_B^2}{e^2 R_T} T\delta T \approx \tilde{g} \mathcal{V}_S \Sigma_S T^5 \tag{S15}$$

$$\frac{\delta T}{T} \approx 1 \;\Rightarrow\; \tilde{g} T^3 \approx T_{x,S}^3 = \frac{k_B^2}{e^2 \rho_\square t_S \Sigma_S}\,. \tag{S16}$$

We assume here that the phonon system is at zero temperature. Here, $\tilde{g}$ is the ratio of suppression factors due to superconductivity, in the tunneling compared to that of e-ph coupling. Moreover, $R_T = \rho_\square/(L_x W)$ is the tunneling resistance where L_x is the junction length, W its width and $\rho_\square$ the square resistivity, and $\mathcal{V}_S = L_x W t_S$ and Σ_S are the volume and the electron-phonon coupling in the superconductor [4], and t_S is the superconductor thickness. At low temperatures (0.2–1.2 K), based on numerical calculations for $\dot{Q}_{\mathrm{e-ph}}/\dot{Q}_{\mathrm{tun}}$, we estimate $\tilde{g} \approx 2(k_B T/\Delta)^2$. Moreover, since T is maintained above the phonon temperature by Joule heating,

$$\Sigma_N T^5 \simeq \rho_N j_H^2 = \rho_N \Big(\frac{V'}{\rho_N L_x}\Big)^2 \;\Rightarrow\; V' = L_x \sqrt{\rho_N \Sigma_N T^5}\,, \tag{S17}$$

where ρ_N, Σ_N are the resistivity and e-ph coupling on the normal side, and j_H the current density of the heating current. Finally, the condition $eV' \ll \Delta$ is equivalent to

$$L_x \ll L_{x,c} = \sqrt{\frac{\Delta^2 \tilde{g}/T^2}{e^2 \rho_N \Sigma_N T_{x,S}^3}} \approx \sqrt{\frac{2\rho_\square t_S \Sigma_S}{\rho_N \Sigma_N}} \approx 100\,\mu\mathrm{m}\,. \tag{S18}$$

Note that the precise value depends on material parameters, also because our estimate for $\tilde{g}$ depends somewhat on values of Γ and spin-flip scattering in the superconductor. The result is however well consistent with the fact that in the experiment of the main text, rectification dominates thermoelectricity.

In the estimates in this section, we have assumed parameter values estimated from our experiment, except for the electron-phonon coupling constants for which literature values are assumed: $\Sigma_S = 2 \times 10^8$ W/m^{3}K^5, $\Sigma_N = 2 \times 10^9$ W/m^{3}K^5, [4] $\rho_\square = 0.39\ \Omega\,\mathrm{mm}^2$, $\rho_N = 3.8 \times 10^{-8}$ Ωm, $t_S = 4$ nm.

---

[1] Stefan Ilić, P. Virtanen, T. T. Heikkilä, and F. Sebastián Bergeret, "Current rectification in junctions with spin-split superconductors," (2021), arXiv:2109.10201.

[2] Tero T Heikkilä, Mikhail Silaev, Pauli Virtanen, and F Sebastian Bergeret, "Thermal, electric and spin transport in superconductor/ferromagnetic-insulator structures," Progr. Surf. Sci. **94**, 100540 (2019).

[3] A. Ozaeta, P. Virtanen, F. S. Bergeret, and T. T. Heikkilä, "Predicted Very Large Thermoelectric Effect in Ferromagnet-Superconductor Junctions in the Presence of a Spin-Splitting Magnetic Field," Physical Review Letters **112**, 057001 (2014).

[4] Francesco Giazotto, Tero T. Heikkilä, Arttu Luukanen, Alexander M. Savin, and Jukka P. Pekola, "Opportunities for mesoscopics in thermometry and refrigeration: Physics and applications," Reviews of Modern Physics **78**, 217–274 (2006).
